# Utility of a novel tapered-tip sheath system for preoperative mapping biopsy of biliary tract cancers

**DOI:** 10.1055/a-2631-7538

**Published:** 2025-07-23

**Authors:** Tomoaki Matsumori, Norimitsu Uza, Kazuhiro Okada, Masahiro Shiokawa, Takahisa Maruno, Yoshihiro Nishikawa, Takeshi Kuwada, Yuya Muramoto, Muneji Yasuda, Hajime Yamazaki, Kojiro Taura, Etsuro Hatano, Yuzo Kodama, Hiroshi Seno

**Affiliations:** 1 34797 Gastroenterology and Hepatology, Kyoto University Hospital, Kyoto City, Japan; 2 12918 Gastroenterology and Hepatology, Kyoto University, Kyoto, Japan; 3 37048 National Hospital Organisation Kyoto Medical Center, Kyoto, Japan; 4 34797 Kyoto University Hospital, Kyoto, Japan; 5 Section of Clinical Epidemiology, Department of Community Medicine, Kyoto University, Kyoto, Japan; 6 566610 Kitano Hospital Medical Research Institute, Osaka, Japan; 7 Division of Gastroenterology, Department of Internal Medicine, Kobe University Graduate School of Medicine, Kobe, Japan; 8 34797 Gastroenterology and Hepatology, Kyoto University Hospital, Kyoto, Japan

**Keywords:** Pancreatobiliary (ERCP/PTCD), Diagnostic ERC, Tissue diagnosis, Cholangioscopy, Strictures

## Abstract

**Background and study aims:**

Preoperative evaluation of biliary tract cancer progression plays a critical role in assessing resectability and in selecting the appropriate surgical procedure. This study aimed to evaluate the utility of a novel tapered-tip sheath system for mapping biopsy to assess the extent of biliary tract cancer.

**Patients and methods:**

This observational, comparative study included 32 patients who were diagnosed with biliary tract cancers and underwent mapping biopsies with the novel tapered-tip sheath system and 21 patients using the conventional methods before the period. Technical success, total biopsy time, number of biopsy specimens, appropriate tissue sampling, adverse events (AEs), and negative surgical margin in case of surgical resection were evaluated.

**Results:**

The following were the respective results for the novel system and conventional methods groups: technical success rates, 73.3% and 48.4% ( *P* = 0.027); total biopsy times, 11.4 and 23.5 minutes ( *P* = 0.043); median number of specimens obtained per procedure, 6 and 3 ( *P* < 0.001); appropriate tissue sampling rates, 86.1% and 67.2% ( *P* < 0.001); AE rates, 2.1% and 0%; and negative surgical margin rates, 90.4% and 78.6%.

**Conclusions:**

Preoperative mapping biopsy using the novel tapered-tip sheath system is a promising option for assessing the extent of biliary tract cancers.

## Introduction

Cholangiocarcinoma has increased in incidence globally over the past few decades, and it exhibits poor prognosis with a 5-year survival rate of 7% to 20% 1 . Despite recent advances in imaging technologies, pathological evaluation based on tissue sampling is crucial for diagnosing cholangiocarcinoma and determining appropriate treatment 2 . Cholangiocarcinoma is characterized by longitudinal extension of the tumor. Therefore, in addition to conventional imaging studies such as computed tomography (CT) 3 , assessment of its superficial extension through mapping biopsy is critical for determining resectability and surgical procedures 4 . Although the necessity of mapping biopsy in biliary tract cancer was previously questioned 5 , subsequent detailed studies have reaffirmed its importance, particularly in the context of early-stage cholangiocarcinoma 6 . Nevertheless, pathological evaluation of biliary tract cancers, including mapping biopsies, presents various challenges.

Unlike biliary stricture biopsies, which include percutaneous, transluminal, and transpapillary approaches, mapping biopsies for assessing superficial extensions are limited to transpapillary procedures. Transpapillary mapping biopsies are mainly performed using fluoroscopy-guided forceps biopsy or peroral cholangioscopy (POCS)-guided biopsy. Fluoroscopy-guided forceps biopsy is often technically challenging in terms of inserting the biopsy forceps directly into the bile duct, passing through the severe cancerous stricture, and selecting the bile duct upstream from the hepatic duct, particularly the left bile duct 7 . Even if a cancerous stricture can be forcefully passed, cancer epithelium may detach upon contact with the biopsy forceps, leading to contamination of the obtained tissue with floating cancer cells and impeding assessment of cancer progression. Moreover, direct and repeated contact of the biopsy forceps with the duodenal papilla or accidental insertion of the biopsy forceps into the pancreatic duct can trigger post-endoscopic retrograde cholangiopancreatography (ERCP) pancreatitis (PEP) 8 9 10 11 12 13 . By contrast, POCS-guided biopsy requires skill and is costly and time-consuming owing to the need for endoscopic sphincterotomy (EST) or irrigation. Furthermore, similar to fluoroscopy-guided forceps biopsy, this method may fail to pass through the cancerous stricture and select its upstream site, owing to the relatively thick and non-tapered shape of the cholangioscope.

Several improvements and development of devices and combinations with other methods have been reported to overcome these challenges, but their ubiquitous use is hindered by their large device diameters or lack of coaxiality 14 15 16 . We previously reported the usefulness of a stent delivery system for mapping biopsy of perihilar cholangiocarcinoma; however, this was not a dedicated device for bile duct biopsy 17 .

We recently developed a novel tapered-tip sheath system (EndoSheather; Piolax Medical Devices, Kanagawa, Japan) for bile duct biopsy ( **Supplementary Fig. 1** ) 18 19 20 . This system has the following features. The tapered tip functions as a dilator, enabling the system to smoothly pass through not only the duodenal papilla but also the cancerous stricture and reach various target bile ducts, including the peripheral bile duct upstream from the cancerous strictures. This system has a coaxial two-layer structure comprising an inner catheter and outer sheath. After removing the inner catheter, the outer sheath serves as a conduit for the biopsy forceps, enabling quick and accurate guidance of biopsy forceps to the target site. In addition, the outer sheath prevents direct and repeated contact of the biopsy forceps with the duodenal papilla and cancerous stricture, which reduces the burden on the duodenal papilla and detachment of cancer epithelium, thereby limiting PEP development and cancer cell contamination, respectively. Instead of small-sized dedicated forceps for POCS, standard-sized biopsy forceps for the gastrointestinal tract can be used, thus allowing for appropriate tissue sampling to improve low diagnostic sensitivity, which is a common challenge for transpapillary bile duct biopsy 21 . This novel system has the potential to be a breakthrough in the mapping biopsy of biliary tract cancers with various challenges. We have reported the usefulness of this system in diagnosing biliary strictures 7 18 .

This study aimed to evaluate the utility of the novel tapered-tip sheath system for the mapping biopsy of biliary tract cancers by comparing it with conventional methods.

## Patients and methods

### Study design and participants

This observational comparative analysis was conducted at Kyoto University Hospital, adhered to the Declaration of Helsinki, and was approved by the Institutional Human Ethics Committee (approval number: R3406). Owing to the retrospective nature of the study, the requirement for written informed consent was waived by the ethics committee. A notification for this study was published on the hospital website to allow patients to opt out of the study.

A total of 32 consecutive patients who underwent ERCP for mapping biopsies using the novel tapered-tip sheath system between July 2020 and August 2021 were included. A total of 21 consecutive patients who underwent mapping biopsies using the conventional methods between October 2015 and June 2020, before commercial availability of the novel tapered-tip sheath system, served as historical controls. All patients were histologically diagnosed with biliary tract cancers, such as cholangiocarcinoma, before mapping biopsy. In patients with extensive biliary stricture, the bile duct with the most intense stricture was defined as the site of principal stricture. Patients were followed-up from first detection of biliary stricture to date of surgery or May 2024.

## Determination of assumed surgical procedures

The assumed surgical procedures were determined by a multidisciplinary team consisting of gastrointestinal endoscopists, surgeons, radiologists, and oncologists, on the basis of imaging studies such as CT and magnetic resonance imaging prior to mapping biopsies. The hilar bile duct was defined as the area bounded by the left hepatic duct edge of the right margin of the portal umbilicus, the right hepatic duct edge of the left margin of the confluence of the anterior and posterior portal vein branches, and the common bile duct edge at the confluence of the bile duct and cystic duct. The peripheral bile duct was defined as the area of the bile duct upstream of the hilar area, and the distal bile duct as the area on the duodenal side of the hilar area.

### Endoscopic procedures

**Fig. 1:**
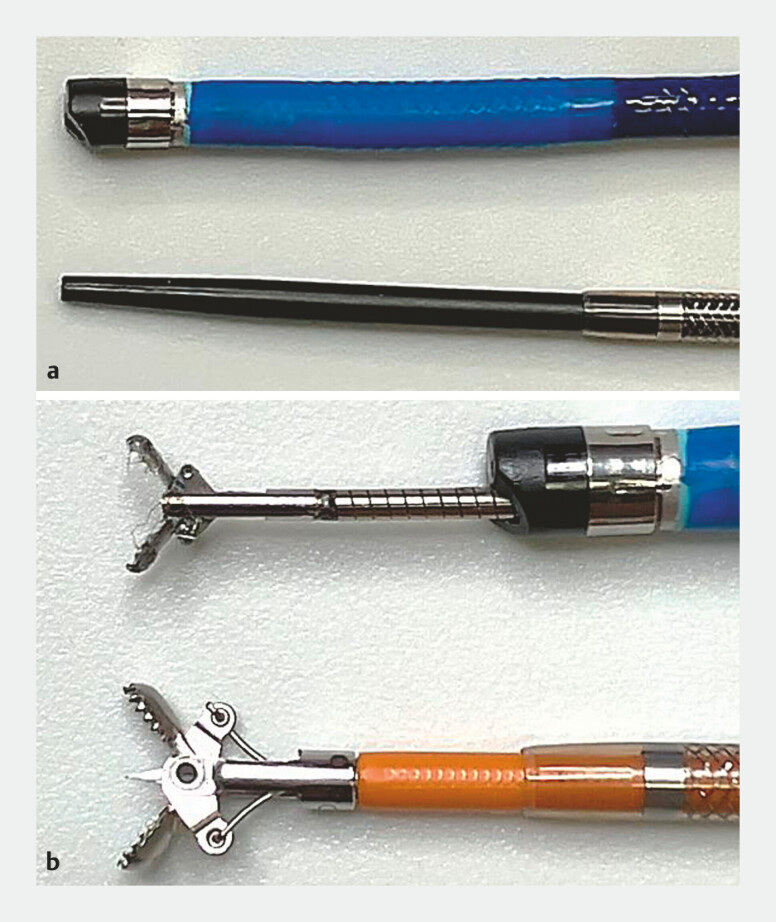
Comparison of tip shape and biopsy forceps between the novel system and peroral cholangioscope. **a** Tip shape of the peroral cholangioscope (POCS) (upper: SpyGlass DS; Boston Scientific Japan, Tokyo, Japan) and novel system (lower: EndoSheather; Piolax Medical Device, Kanagawa, Japan). **b** Small cup biopsy forceps dedicated to the POCS (upper: Spy Bite Max; Boston Scientific Japan) and standard-sized cup biopsy forceps with a needle loaded in the novel system (lower: Radial jaw 4P Pediatric Biopsy Forceps; Boston Scientific Japan).

**Fig. 2:**
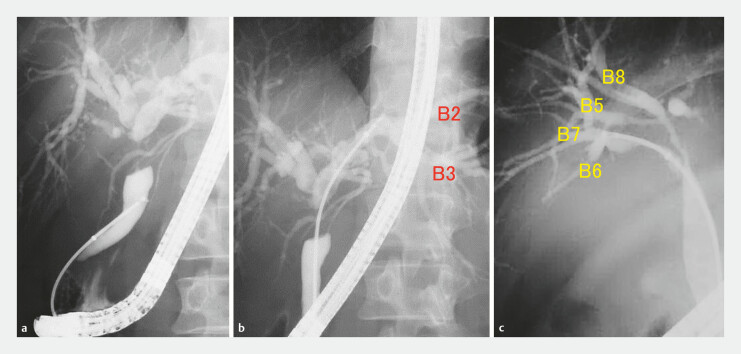
Images of fluoroscopy-guided mapping biopsy using the novel tapered-tip sheath system. **a,b** Mapping biopsy at the distal bile duct ( **a** ) and B2/3 confluence ( **b** ) in a patient with perihilar cholangiocarcinoma for whom right lobectomy was assumed. **c** Mapping biopsy at the top of the posterior bile duct branch, in a patient with perihilar cholangiocarcinoma for whom left hepatic trisegmentectomy was assumed.

ERCP was performed in the standard fashion using a side-viewing duodenoscope (TJF-260V; Olympus Medical System, Tokyo, Japan). Anatomical features and biliary strictures were evaluated by cholangiography and intraductal ultrasonography, followed by biliary stricture biopsy to make a definitive diagnosis. Some patients were referred to our hospital after these processes were performed at other hospitals. Considering the patient burden, mapping biopsy was conducted on a separate day from the biliary stricture biopsy using either the novel system or conventional methods: fluoroscopy-guided forceps biopsy or POCS-guided biopsy. Biopsy forceps with a standard-sized cup (Radial jaw 4P Pediatric Biopsy Forceps; Boston Scientific Japan, Tokyo, Japan), commonly used for gastrointestinal diseases, was utilized with the novel system or during fluoroscopy-guided forceps biopsy. Dedicated biopsy forceps with a small cup for POCS (SpyBite; Boston Scientific Japan, Tokyo, Japan) was used during POCS-guided biopsy (SpyGlass DS; Boston Scientific Japan, Tokyo, Japan or CHF B260; Olympus Medical System, Tokyo, Japan) ( Fig. 1 , **Supplementary Fig. 2** ). EST was performed in cases involving POCS, difficult insertion of biopsy forceps into the bile duct, or metal stent placement. In the conventional group, criteria for selecting between fluoroscopy-guided biopsy using standard biopsy forceps and POCS-guided biopsy were as follows: biopsy was initially attempted by directly inserting standard forceps into the bile duct under fluoroscopic guidance. In cases in which this approach was not feasible, when there was a discrepancy between the extent of tumor spread suggested by imaging and the mapping biopsy results under fluoroscopy, or when pre-procedure imaging such as CT, endoscopic ultrasound, cholangiography, and intraductal ultrasonography suggested presence of papillary tumors—which are often associated with extensive superficial spread and presumed to involve soft strictures—POCS-guided biopsy was selected. To improve diagnostic performance, up to three biopsy specimens were tried to obtain tissue from the resection line of the bile duct on the basis of the planned surgical procedures according to previous reports ( Fig. 2 ) 22 .

### Pathological definition

Three cytopathologists specializing in pancreaticobiliary diseases evaluated biopsy specimens. Specimens containing cancer cells or normal epithelial cells with stroma that could be evaluated histopathologically were defined as appropriate tissue samples. Specimens described as “suspected carcinoma” and "“carcinoma” in the histological findings were classified as malignant.

### Outcome measurements

We compared the following outcome measures between the two groups: technical success, total biopsy time, number of biopsy sites and specimens, appropriate tissue sampling, radiation exposure time, history of EST, adverse events (AEs), and negative surgical margins in cases with surgical resection.

The target site for the mapping biopsy was defined as the resection line for the assumed surgical procedures. Technical success was defined as the ability to obtain at least one grossly visible tissue sample from all target sites. Total biopsy time was defined as time from the first biopsy to completion of the last biopsy, and was calculated from the examination database and video recordings. Total radiation exposure time was calculated from data recorded by the x-ray fluoroscopy system (DREX-UI80/06; Toshiba, Tochigi, Japan). Consensus guidelines were used to define, grade, and treat ERCP-related AEs 23 .

### Statistical analyses

Patient data were collected from the medical records, and statistical analyses were conducted using GraphPad Prism 7 (GraphPad Software, California, United States) and Excel software (Microsoft Corp., Washington, United States). Continuous variables are presented as numbers, medians, and ranges, and comparisons were performed using the Mann-Whitney U test. Categorical variables are presented as numbers and percentages, and comparisons were performed using the chi-square test and Fisher’s exact test. Differences were considered statistically significant at *P* < 0.05.

## Results

Overall, 53 patients with biliary tract cancers were enrolled in this study. Preoperative mapping biopsy was performed on 32 patients by using the novel device and in 21 patients by using the conventional methods. No substantial differences in patient characteristics, such as final diagnosis and stricture site, were observed between the two groups ( Table 1 ).

**Table:** **Table 1** Patient characteristics.

	Novel system	Conventional method	P value
**Number of patients, n**	32	21	
**Age, median (range)**	73 (40–87)	70 (46–83)	0.208
**Sex, male:female**	29:3	14:7	0.069
**Final diagnosis, n**			
Distal cholangiocarcinoma	8	9	0.181
Hilar cholangiocarcinoma	16	7	0.345
Intrahepatic cholangiocarcinoma	6	4	0.883
Gallbladder carcinoma	0	1	0.333
Ampulla of Vater carcinoma	2	0	0.525
**Stricture site, n**			
Distal	10	10	0.229
Hilar	16	7	0.345
Peripheral	6	4	0.883

Outcomes of the mapping biopsy procedures are summarized in Table 2 . Overall, 45 mapping biopsies were performed in the novel system group and 31 (14 fluoroscopy-guided forceps biopsies and 17 POCS-guided biopsies) in the conventional method group. Overall technical success rates were 73.3% (33/45) and 48.4% (15/31) in the novel system and conventional method groups, respectively ( *P* = 0.027). Within the conventional methods group, technical success was observed in 28.6% (4/14) and 64.7% (11/17) of the fluoroscopy- and POCS-guided biopsy groups, respectively ( **Supplementary Table 1** ). Considering technical failure, most cases in the novel system group had difficulty in biopsy of the distal bile ducts despite the biopsy forceps reaching the target site. The fluoroscopy-guided biopsy group of the conventional method group faced challenges in delivering the biopsy forceps to various bile ducts, and the POCS-guided biopsy group had difficulty in advancing the cholangioscope beyond the cancerous stricture.

**Table:** **Table 2** Outcomes of mapping biopsy procedures.

	Novel system	Conventional method	*P* value
**Number of procedures**	45	31	N/A
**Number of procedures per patient, median (range)**	1 (1–4)	1 (1–4)	0.908
**Technical success, % (n/N)**	73.3 (33/45)	48.4 (15/31)	0.027
**Reasons for technical failure, n**			
Inability to perform biopsy at the target site, n			
Distal bile duct	10	5	
Left peripheral bile duct	1	5	
Right peripheral bile duct	1	3	
Inability to guide the POCS to the target site, n	0	3	
**Total biopsy time, minutes, median (range)**	11.4 (0.9–40.1)	23.5 (3.8–89.3)	0.043
**Number of biopsy sites, median (range)**	2 (2–6)	1 (1–6)	0.026
**Number of biopsy specimens per procedure, median (range)**	6 (1–12)	3 (1–13)	< 0.001
**Total radiation exposure time, minutes, median (range)**	25 (7–54)	31 (12–90)	0.003
**EST, % (n/N)**	31.1 (14/45)	71.0 (22/31)	0.001
Prior EST, n	12	9	
Newly EST, n	2	13	
**Pancreatic stent placement during procedure, % (n/N)**	0 (0/45)	0 (0/31)	N/A
**Adverse events, % (n/N)**	2.2 (1/45)	0 (0/31)	N/A
Acute pancreatitis			
Mild	1	0	
EST, endoscopic sphincterotomy; N/A, not applicable; POCS, peroral cholangioscopy.			

Total biopsy time was significantly shorter in the novel system group than in the conventional method group (11.4 minutes [range 0.9–40.1] vs. 23.5 [range 3.8–89.3]; *P* = 0.043). Analysis within the conventional method group showed that the POCS-guided forceps biopsy required a longer biopsy time than the fluoroscopy-guided forceps biopsy (54.5 [19.7–89.3] minutes vs. 7.8 [3.8–24.7], *P* = 0.081) ( **Supplementary Table 1** ).

The number of biopsy sites and specimens obtained per procedure was significantly higher in the novel system group than in the conventional method group (2 [range: 2–6] vs. 1 [range 1–6]; *P* = 0.026 and 6 [range: 1–12] vs. 3 [range 1–13]; *P* < 0.001, respectively).

The novel and conventional groups had 274 and 116 biopsy specimens, respectively ( Table 3 ). The overall appropriate tissue sampling rate was significantly higher in the novel system group than in the conventional method group (86.1% [236/274] vs. 67.2% [78/116]; *P* < 0.001) and was higher in the hilar and peripheral bile ducts (94.2% [49/52] vs. 61.3% [19/31]; *P* < 0.001 and 82.2% [125/152] vs. 62.5% [35/56]; *P* = 0.003, respectively).

**Table:** **Table 3** Biopsy specimens and tissue sampling using the novel system and conventional method

	Novel system	Conventional method	*P* value
**Total number of biopsy specimens, n**	274	116	
**Appropriate tissue sampling, % (n/N)**	86.1 (236/274)	67.2 (78/116)	< 0.001
Distal	88.6 (62/70)	82.8 (24/29)	0.651
Hilar	94.2 (49/52)	61.3 (19/31)	< 0.001
Peripheral	82.2 (125/152)	62.5 (35/56)	0.003

Total radiation exposure time was significantly shorter in the novel system group than in the conventional method group (25 minutes [range 7–54] vs. 31 [range 12–90], *P* = 0.003) and tended to be longer in the POCS-guided biopsy group than in the fluoroscopy-guided biopsy group among the conventional methods (36 minutes [range 23–90] vs. 20 [range 12–90], *P* = 0.096) ( **Supplementary Table 1** ).

EST was performed in 31.1% of patients (14/45) in the novel system group and 71% of patients (22/31) in the conventional method group ( *P* = 0.001), with 21 of 36 procedures already performed prior to mapping biopsy. Regarding cases in which EST was newly performed at our institution, two of 14 cases in the novel system group were for stenting and not for biopsy. Meanwhile, in the conventional method group, EST was newly performed in 13 cases in which the insertion of biopsy forceps or cholangioscope was challenging. No differences in AEs were noted between the groups (2.2% [1/45] vs. 0% [0/31]).

Surgery was performed in 65.6% (21/32) and 66.7% of patients (14/21) in the novel system and conventional method groups, respectively ( *P* = 0.827) ( Table 4 ).

**Table:** **Table 4** Details of resected cases.

	Novel system	Conventional method	*P* value
**Surgical cases, % (n/N)**	65.6 (21/32)	66.7 (14/21)	0.827
**Change of the assumed surgical procedure, % (n/N)**	38.1 (8/21)	42.9 (6/14)	0.969
**Surgical procedure. N**			
Left lobectomy	9	2	0.066
Pancreatoduodenectomy	6	5	0.852
Right hepatopancreatoduodenectomy	3	1	0.951
Right lobectomy	2	3	0.357
Left hepatic trisegmentectomy	1	0	0.817
Left hepatopancreatoduodenectomy	0	1	0.389
Right hepatic trisegmentectomy	0	2	0.144
**Negative surgical margin, % (n/N)**	90.4 (19/21)	78.6 (11/14)	0.369

In addition, reasons for unresected case are presented in **Supplementary Table 2** . In both groups, there were no significant differences in the change of the assumed surgical procedure according to results of the mapping biopsy (38.1% [8/21] vs. 42.9% [6/14]; *P* = 0.969) or negative surgical margin (90.4% [19/21] vs. 78.6% [11/14]; *P* = 0.369).

## Discussion

Pathological evaluation of longitudinal extension of biliary tract cancer is essential to determine resectability and appropriate surgical interventions 4 6 24 . However, conventional mapping biopsy methods pose various challenges. This study shows that the novel tapered-tip sheath system is a promising tool for mapping biopsy to evaluate longitudinal extension of biliary tract cancer.

Unlike biliary stricture biopsies, the access route for mapping biopsies is limited to the transpapillary approach. Two crucial steps are involved in pathological evaluation of bile ducts using transpapillary approaches: delivery of diagnostic devices to the target bile duct and appropriate tissue collection for pathological assessment. When mapping biopsy, various bile ducts, including upstream bile ducts beyond the cancerous stricture as well as the distal side of the primary lesion, should be reached and evaluated. We focused on the initial step of bile duct evaluation (i.e., diagnostic device delivery) and showed that greater technical success was achieved in the novel system group than in the conventional method group. This may be attributed to the tapered tip-shape and thin outer diameter of the novel system, which facilitated access to various target bile ducts and enabled delivery of standard-sized biopsy forceps to the bile duct of interest via the outer sheath after removing the inner catheter ( Fig. 1 ). A comparative study of mapping biopsies using a stent delivery system with a coaxial two-layer structure and a POCS for biliary tract cancer reported a 100% technical success rate using the stent delivery system 25 ; this result was different from our outcome using the novel device with a similar structure (100% vs. 73.3%). This discrepancy may be attributed to differences in definitions of technical success. Although our study defined technical success as reaching the target site and obtaining at least one grossly visible tissue fragment from all target sites, the previous study considered technical success as the biopsy forceps reaching the target bile duct. According to the definition by Takeda et al., the technical success rate would be 100% even for the novel system group in our study, because the biopsy forceps could reach the target bile duct even in cases of technical failure.

In this study, technical failures were observed in 26.6% of procedures (12/45) using the novel system, particularly in the distal bile duct. These failures occurred despite delivery of biopsy forceps because the biopsy forceps slipped easily and failed to efficiently collect tissue in the normal mucosa subjected to the mapping biopsy owing to parallel alignment of the biopsy forceps and bile duct axes in the distal bile duct. In the remaining two cases in the novel system group, biopsy forceps could be delivered to the left and right peripheral bile ducts; however, narrowness of the bile ducts hindered opening of the biopsy forceps. By contrast, the axes of the biopsy forceps and bile duct were not aligned for fluoroscopy-guided forceps biopsy of the conventional method group, which prevented delivery of the biopsy forceps to various bile ducts, including the left bile duct. Although the POCS-guided biopsy group had an advantage in evaluating the bile ducts distal to the cancerous stricture, the large diameter and non-tapered shape of the cholangioscope made it difficult to pass through the cancerous stricture and evaluate upstream bile ducts in some cases. Collectively, these results suggest that mapping biopsy using the novel system is useful in the first step of bile duct evaluation (diagnostic device delivery) and has challenges in the second step (appropriate tissue collection). Conversely, conventional methods face issues in the first step rather than the second step of pathological evaluation of the bile ducts. Greater technical success and appropriate tissue collection can be expected in the novel system group if improvements to biopsy forceps, such as prevention of slippage, can be achieved. Indeed, use of biopsy forceps with a needle has recently enabled biopsies to be performed without slipping ( **Supplementary Fig. 2** ).

One of the challenges of biliary stricture biopsy is its low diagnostic sensitivity 26 27 28 , which may also be applicable to mapping biopsy. Reportedly, factors affecting diagnostic sensitivity of bile duct biopsy include volume and number of samples collected 21 22 . The novel system can theoretically yield a larger tissue volume because it allows for use of larger biopsy forceps than the dedicated forceps for POCS. In addition, because the outer sheath functions as a conduit for the biopsy forceps, repeated tissue sampling can be performed within a short period and a large number of tissues can be obtained. Thus, this novel system is expected to improve diagnostic sensitivity in terms of sample volume and number. In fact, a large number of samples appropriate for pathological evaluation were obtained from the novel device group ( Table 3 ). This result was particularly evident in the hilar region and peripheral bile ducts, which benefited from easy delivery of the biopsy forceps using the novel device.

Compared with biliary stricture biopsies, mapping biopsies typically require longer examination and biopsy times, owing to the large number of biopsy sites. However, few studies have examined time required for mapping biopsies. In this study, mapping biopsies with the novel system were completed quicker, even though more specimens could be obtained at more sites. Prolonged transpapillary procedures carry risk of ERCP-related AEs, including PEP 11 29 . Furthermore, prolonged procedures require higher levels of sedation and expose both patients and physicians to excessive radiation 30 31 . This study showed that the novel system group had shorter procedure times and less radiation exposure. These findings can be attributed to the outer sheath, which serves as a conduit for the biopsy forceps after removal of the inner catheter and facilitates quick and easy navigation of the biopsy forceps to the target bile duct. Therefore, the novel device is a useful tool for ensuring safety because it shortens examination time and reduces ERCP-, sedation-, and radiation-related AEs.

Despite the complex and invasive nature of mapping biopsy, limited reports have described AE specific to the procedure 17 25 . Herein, no differences in AEs were observed between the two groups. Notably, incidence of PEP did not increase in the novel device group, despite the significantly higher number of cases in which EST was not performed. A retrospective study by Tamura et al. 13 and our recent prospective trial 32 suggest that fluoroscopy-guided forceps biopsy without EST may be a risk factor for developing PEP. Although EST may facilitate insertion of biopsy forceps and cholangioscope, thereby reducing risk of PEP, it carries risk of bleeding and perforation. Thus, no increased risk of developing PEP with transpapillary bile duct biopsy without EST may be a major advantage of this device. The novel device has the potential to provide a safe and reliable route for the efficient delivery of biopsy forceps even without performing EST. Furthermore, the outer sheath of the novel system avoids direct and frequent contact between the biopsy forceps and the duodenal papilla and prevents accidental insertion of the biopsy forceps into the pancreatic duct, thereby reducing PEP development.

This study compared the novel system with conventional methods consisting of fluoroscopy- and POCS-guided biopsy. In addition, POCS-guided biopsy included video cholangioscopy using a mother-baby system and single-operator cholangioscopy (SOCS). Consequently, each patient incurred a different device cost per examination. The SOCS-based procedure costs approximately USD 3,600 for the cholangioscope and dedicated biopsy forceps alone, excluding the digital imaging system. By contrast, the novel method costs approximately USD 290, including the biopsy forceps. Considering safety, maneuverability, diagnostic performance, and device cost, it is crucial to select an optimal method on the basis of feasibility at each facility.

In the resected cases, no significant difference was observed between the two groups in the rate of surgical procedure modification based on results of mapping biopsy. In all cases in which a change in surgical procedure was required, the reason was that mapping biopsy revealed either a greater tumor extent than initially predicted by imaging studies or overestimation of tumor spread on imaging. In some cases, this allowed for avoidance of unnecessarily extensive and invasive surgical procedures ( **Supplementary Table 3** ). One possible reason for the relatively high frequency of surgical plan modifications is the retrospective nature of this study, in which the timing of surgical planning varied among cases. One contributing factor may have been that, in some cases, the anticipated surgical procedure was determined solely based on CT findings. For future studies aiming at more detailed analyses and standardization of both the timing of surgical planning and the imaging modalities used for decision-making will be essential.

Based on the results of this study, we believe that performing mapping biopsy using the novel tapered-tip sheath system as the initial approach may be beneficial in terms of simplicity, tissue sampling, reduced procedure time, and cost. In cases in which mapping biopsy with the novel system is technically challenging for certain locations, or when there is a discrepancy between the extent of tumor spread suggested by imaging and mapping biopsy results using the novel system, POCS-guided biopsy may be a suitable alternative. On the other hand, for papillary tumors that are expected to have extensive superficial spread and soft strictures, POCS-guided biopsy—which also allows for direct visualization of the epithelium—may be considered the first device for mapping biopsy. However, further studies are needed to validate these considerations. To verify which is the best strategy for mapping biopsy, we are currently planning a prospective comparative study to evaluate the performance of biopsy using the novel system versus POCS-guided biopsy.

From the analysis of the study results, there were cases in which the quantity of tissue samples obtained was insufficient, potentially due to limitations of the biopsy forceps. To improve accuracy of tissue acquisition, modifications to the novel system are currently underway to enable insertion of larger biopsy forceps capable of obtaining more substantial specimens. In addition, biopsy forceps specifically designed to facilitate easier and more efficient tissue sampling from the bile duct are also under development.

This study had some limitations. First, this was a retrospective, comparative study with a small sample size conducted at a single center, and there may have been temporal and patient selection biases between the two groups. Given recent advancements in POCS technology, the results obtained from the group that underwent POCS-guided biopsy may differ from those that would be achieved using the current POCS systems. In addition, because use of the novel tapered-tip sheath system is associated with a learning curve, the results may vary depending on the current level of adoption and familiarity with the system. To address these bias issues, we consider that dividing patients from the same time period into two groups for comparison would yield more accurate results. A multicenter prospective study is currently being planned to compare the diagnostic performance of mapping biopsies using a novel device with those performed under POCS. Second, this study could not determine which method was superior for predicting negative surgical margins because some cases used multiple methods. Future parallel-group comparative studies are required to validate the accuracy of each method selected for mapping biopsies of biliary tract cancers.

## Conclusions

In conclusion, the novel tapered-tip sheath system enables easy and safe guidance of biopsy forceps to the target bile duct and may be a promising device for mapping biopsies to assess longitudinal extension of biliary tract cancers.

## Supplementary Material

Supplementary Material Zusatzmaterial
