# Supplementary material for: Utility of a novel tapered-tip sheath system for preoperative mapping biopsy of biliary tract cancers
Source: Endosc Int Open. 2025 Jul 23;13:a26317538. doi: 10.1055/a-2631-7538 (PMC12303023; doi:10.1055/a-2631-7538)
Supplement: Supplementary file 1 — Supplementary Material [file 10-1055-a-2631-7538_26347236.pdf]

Supplementary Table 1 Details of conventional method.

	Fluoroscopy-guided biopsy	POCS-guided biopsy	*P* value
**Number of procedures**	14	17	
**Location of stricture, n**			
Distal	7	10	0.898
Hilar	7	4	0.248
Peripheral	0	3	0.232
**Technical success, % n/N**	28.6 (4/14)	64.7 (11/17)	0.1001
**Total biopsy time, minutes, median (range)**	7.8 (3.8–24.7)	54.5 (19.7–89.3)	0.081
**Number of biopsy site, median (range)**	1 (1–2)	2 (1–6)	0.160
**Number of biopsy specimens per procedure, median (range)**	2 (2–5)	5 (1–13)	0.075
**Total number of biopsy specimens, n**	41	75	
**Appropriate biopsy sampling, % (n/N)**	73.2 (30/41)	64.0 (48/75)	0.314
Distal	81.5 (22/27)	100 (2/2)	N/A
Hilar	75.0 (3/4)	59.3 (16/27)	0.967
Peripheral	50.0 (5/10)	65.2 (30/46)	0.589
**Total radiation exposure time, minutes, median (range)**	20 (12–90)	36 (23–90)	0.096
**EST, % (n/N)**	35.7 (5/14)	100 (17/17)	<0.001
Prior EST, n	2	7	
Newly EST, n	3	10	

EST, endoscopic sphincterotomy; N/A, not applicable; POCS, peroral cholangioscopy.

Endosc Int Open | DOI 10.1055/a-2631-7538 | © The Author(s).

Supplementary Table 2 Reasons for unresected cases.

	Novel system	Conventional method
**Unresected cases, N**	11	7
**Reasons for unresectability, N**		
Medically inoperable	4	3
Extensive tumor spread detected by mapping biopsy	4	1
Locally advanced disease identified on preoperative evaluation	1	2
Lymph node metastasis confirmed by intraoperative frozen section	2	0
Withdrawal of consent for surgery	0	1

Endosc Int Open | DOI 10.1055/a-2631-7538 | © The Author(s).

Supplementary Table 3 Details about change in surgical procedure.

**Prior to change**	**After change**	**Novel system**	**Conventional method**
Right lobectomy	Right hepatopancreatoduodenectomy	2	1
Right hepatopancreatoduodenectomy	Pancreatoduodenectomy	2	0
Left hepatopancreatoduodenectomy	Left lobectomy	2	1
Right lobectomy	Lefthepatic trisegmentectomy	1	0
Pancreatoduodenectomy	Right hepatopancreatoduodenectomy	1	0
Right lobectomy	Pancreatoduodenectomy	0	2
Right hepatopancreatoduodenectomy	Left hepatopancreatoduodenectomy	0	1
Right lobectomy	Right hepatic trisegmentectomy	0	1

Endosc Int Open | DOI 10.1055/a-2631-7538 | © The Author(s).

Supplementary Fig. 1 Images of a novel tapered-tip sheath system. **a** Overview of the novel tapered-tip sheath system. **b** The coaxial two-layer structure comprising an inner catheter with a tapered tip and an outer sheath with a radiopaque marker. **c** A guidewire inserted into the inner catheter of the novel system. **d** Tip of the outer sheath after removing the inner catheter and guidewire. **e** The outer sheath loaded with biopsy forceps.

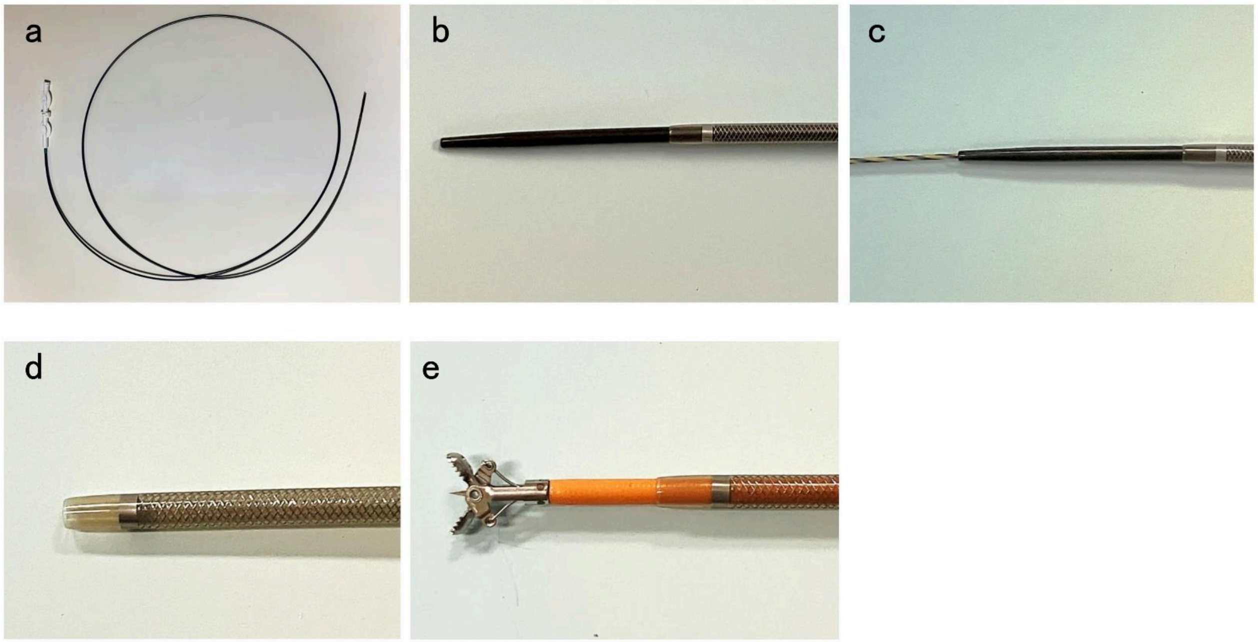

Endosc Int Open | DOI 10.1055/a-2631-7538 | © The Author(s).

Supplementary Fig. 2 Comparison of standard-sized biopsy forceps and peroral cholangioscope-dedicated biopsy forceps. **a** Standard-sized cup biopsy forceps (left: Radial jaw 4P Pediatric Biopsy Forceps; Boston Scientific Japan) and a peroral cholangioscope (POCS)-dedicated biopsy forceps (right: Spy Bite Max; Boston Scientific Japan). **b** Standard-sized cup biopsy forceps (left), standard-sized cup biopsy forceps with a needle (middle), and small cup biopsy forceps for POCS (right).

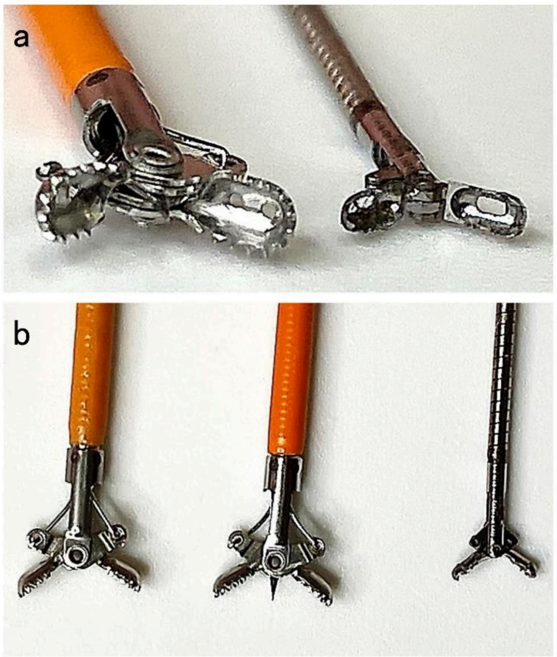

Endosc Int Open | DOI 10.1055/a-2631-7538 | © The Author(s).
